# Factors associated with motivation and hesitation to work among health professionals during a public crisis: a cross sectional study of hospital workers in Japan during the pandemic (H1N1) 2009

**DOI:** 10.1186/1471-2458-10-672

**Published:** 2010-11-04

**Authors:** Hissei Imai, Kunitaka Matsuishi, Atsushi Ito, Kentaro Mouri, Noboru Kitamura, Keiko Akimoto, Koichi Mino, Ayako Kawazoe, Masanori Isobe, Shizuo Takamiya, Tatsuo Mita

**Affiliations:** 1 Department of Psychiatry, Kobe City Medical Center General Hospital, 4-6 Nakamachi Minatojima Chuo-ku, Kobe, Japan; 2 Department of Psychiatry, Kobe City Medical Center West Hospital. 2-4 Ichibancho Nagata-ku, Kobe, Japan; 3 Department of Psychiatry, Nishi-Kobe Medical Center, 5-7-1 Kojidai Nishi-ku, Kobe, Japan

## Abstract

**Background:**

The professionalism of hospital workers in Japan was challenged by the pandemic (H1N1) 2009. To maintain hospital function under critical situations such as a pandemic, it is important to understand the factors that increase and decrease the willingness to work. Previous hospital-based studies have examined this question using hypothetical events, but so far it has not been examined in an actual pandemic. Here, we surveyed the factors that influenced the motivation and hesitation of hospital workers to work in Japan soon after the pandemic (H1N1) 2009.

**Methods:**

Self-administered anonymous questionnaires about demographic character and stress factors were distributed to all 3635 employees at three core hospitals in Kobe city, Japan and were collected from June to July, 2009, about one month after the pandemic (H1N1) in Japan.

**Results:**

Of a total of 3635 questionnaires distributed, 1693 (46.7%) valid questionnaires were received. 28.4% (N = 481) of workers had strong motivation and 14.7% (N = 249) had strong hesitation to work. Demographic characters and stress-related questions were categorised into four types according to the odds ratios (OR) of motivation and hesitation to work: some factors increased motivation and lowered hesitation; others increased motivation only; others increased hesitation only and others increased both motivation and hesitation. The strong feeling of being supported by the national and local governments (Multivariate OR: motivation; 3.5; CI 2.2-5.4, hesitation; 0.2; CI 0.1-0.6) and being protected by hospital (Multivariate OR: motivation; 2.8; CI 2.2-3.7, hesitation; 0.5; CI 0.3-0.7) were related to higher motivation and lower hesitation. Here, protection included taking precautions to prevent illness among workers and their families, providing for the care of those who do become ill, reducing malpractice threats, and financial support for families of workers who die on duty. But 94.1% of the respondents answered protection by the national and local government was weak and 79.7% answered protection by the hospital was weak.

**Conclusions:**

Some factors have conflicting effects because they increase both motivation and hesitation. Giving workers the feeling that they are being protected by the national and local government and hospital is especially valuable because it increases their motivation and lowers their hesitation to work.

## Background

The professionalism of hospital workers was challenged by the pandemic (H1N1) 2009. To maintain the function of hospitals under high risk conditions in the future, it is important to clarify the factors that promote or hinder a professional attitude in actual situations.

Historically, the professionalism of medical workers has been tested by various events such as HIV, Ebola hemorrhagic fever, the Tokyo sarin gas attack, SARS and so on. Among these events, SARS raised the question of how professionals should respond in public emergencies. SARS spread to 26 countries, where it infected 8096 people and killed 774 (mortality rate: 9.6%). Most hospitals continued to serve the public, but at least one hospital in China ceased to function because of mass absence of its workers [1]. Many people in the public were afraid of what would happen if infections like SARS occurred on a pandemic scale.

After the SARS crisis, various studies were carried out, in which hospital workers were asked how they would respond to a hypothetical pandemic infection. In Germany, 28% of nurses, doctors, medical students and hospital officials answered that they might be absent from work during a pandemic to protect themselves and their families [2]. In the United States, 46.2% of local public health workers reported that they would probably not work during a future influenza pandemic [3] and 21.7% of health care employees would be unwilling to work during a SARS pandemic [4]. In Singapore, 27.7% of primary care physicians would not look after patients infected with avian influenza [5]. In Canada, 21% of family physicians indicated that they would be unwilling to help in a pandemic infection if their help was requested by the public health department [6]. Overall around 20 or 30% of health care-related workers showed a hesitation to work during a future infection pandemic regardless of their culture.

On June 11, 2009, WHO declared the H1N1 influenza infection a pandemic. On May 16, our hospital admitted the first patient that had been domestically infected with the H1N1 influenza virus in Japan. In the following two weeks, 1687 people who suspected that they had H1N1 influenza infection came to our hospitals and were released as outpatients and an additional 144 patients who we suspected as having H1N1 were admitted. Of these, 49 were diagnosed as having an H1N1 influenza infection after they were admitted. Kobe City Medical Center General Hospital had 122 admissions who were suspected to be H1N1-positive, including 31 patients who were subsequently diagnosed with H1N1. Kobe City Medical Center West Hospital had 22 admissions including 18 patients diagnosed with H1N1 afterward. The peak was May 17 and the number of patients coming to our outpatient unit for H1N1 infection on that day was 211. On May 27, the mayor of Kobe city declared the emergency had subsided. On June 3, the outpatient unit for H1N1 infection was closed.

To the best of our knowledge, no studies have evaluated hospital workers' willingness to work and the factors that influence their decisions in a real pandemic.

Individuals interacting within a social setting are known to be subject to intrinsic and extrinsic motivation, and are often manipulated or managed to strategically meet societal and/or organizational goals [7,8]. Professionals, who are traditionally granted a high degree of autonomy, may be particularly sensitive to incentives and disincentives, of whatever nature [9]. A hospital-based study suggested that the willingness of workers to respond to an influenza pandemic is powerfully influenced by their perceptions of threat and efficacy [10]. Professional conduct of physicians is affected by incentives and disincentives [11-16]. From these points of view, the willingness to work is thought to be a function of the conflicting factors of motivation (incentives), and hesitation (disincentives). To maintain willingness of hospital workers and improve hospital function in critical situations, it is important to understand the factors that motivate hospital workers to work and that discourage them from working. After our experience with the H1N1 influenza pandemic, we investigated the attitudes of workers in Kobe area hospitals about willingness to work in a pandemic and the factors that influence them by using questionnaire.

## Methods

This survey was approved by the Kobe City Medical Center General Hospital Ethical Review Board. Participation in this survey was voluntary.

We conducted the study at Kobe City Medical Center General Hospital (912 beds), Kobe City Medical Center West Hospital (358 beds) and Nishi-Kobe Medical Center (500beds), which compose Kobe City Hospital Organization and are tertiary teaching hospitals in Kobe city. All three hospitals accepted H1N1 influenza patients starting March 16, 2009, when the first domestically infected patient visited Kobe City Medical Center General Hospital. Paper-based self-administered anonymous questionnaires were personally handed to all employees or placed in their mail boxes from June 22, 2009 and were collected from collecting boxes in the participating hospitals till July 31, 2009, which is about one month after the peak of the H1N1 outbreak in Kobe city. When this survey was conducted, the level of the pandemic was phase 6 in the world and the number of patients in Japan was growing, but the alert to the infection was downgraded as information accumulated that the virulence was not strong. By June 8, 2009, our hospitals returned to their normal practice.

### Survey content

The questionnaire explained its purpose and stated that the results would be published, and respondents would remain anonymous. The first item asked for approval to use the responses in the survey. Answers without this approval were omitted from the analysis. The questionnaire contained 20 items that addressed sociodemographic characteristics, perceived stress associated with the H1N1 event, and motivation and hesitation to work during the event (Additional file 1).

The personal characteristics included gender, age, job and working place (the ward for H1N1, the outpatient department for H1N1, emergency outpatient unit, headquarter and others).

The stress-related questions were as follows: anxiety about being infected; anxiety about infecting family; anxiety of being infected during commuting; lack of knowledge about infectiosity and virulence; lack of knowledge about prevention and protection; feeling of being protected by national and local government; feeling of being protected by hospital (the protection include taking all reasonable precautions to prevent illness, providing for the care of those who do become ill, reducing malpractice threats for those working in high-risk emergency situations and providing reliable compensation for the families of those who die while fulfilling this duty and attenuating the duty of hospital workers not to become a patient him or herself and so on); anxiety about compensations; burden of increase quantity of work; burden of change of quality of work; physical exhaustion; mental exhaustion; insomnia; elevated mood; feeling of being avoided by others; feeling of being isolated; feeling of having no choice but to work due to obligation; burden of child care including lack of nursery. These are the essential items from previous studies on SARS [17,18] and hypothetical infection pandemics [2,3,5,19] and hypothetical symptoms during crises. The respondents used a 4-point Likert scale (0; "never", 1; "rarely", 2; "sometimes", 3; "always") to respond to the questions about how often they felt about the 18 items. The responses of how often they felt motivation and hesitation to work were also scored by a 4-point Likert scale as above.

The jobs of hospital workers were classified into three categories: (1) clinical staff (doctors and nurses); (2) clinical technical/support staff (radiological technologists, clinical laboratory technicians, pharmacists, dieticians, social workers, physical therapists, occupational therapists and speech therapists); and (3) non-clinical staff (office workers, clinical clerks, guards, janitors and others). Working places were categorised into the high-risk places (the ward and the outpatient department for H1N1 influenza infection, emergency outpatient unit and headquarter) and the low-risk places (others). We were unable to determine how many workers in high risk places actually came in contact with H1N1 patients, but all such workers could have come in contact with H1N1 patients and they recognized this.

### Data analysis

Responses to the stress-related questions and motivation and hesitation to work were dichotomized into responses with a score two or less (weak) and all other (strong) responses. Bivariate and multivariate logistic regression models (adjusted for age, gender, job and working place) were used to compute odds ratios (OR) to evaluate the association of personal characteristics variables and stress-related items with self-described motivation and hesitation to work. SPSS (17.0J: Tokyo) was used for data capturing and analysis.

## Results

We sent out a total of 3635 questionnaires. We received a total of valid 1995 questionnaires (54.9%). The breakdown of the responses is as follows: we received 1081 out of 1625 (66.5%) from Kobe City Medical Center General Hospital, 313 out of 775 (40.4%) from Kobe City Medical Center West Hospital and 601 out of 1235 (48.7%) from Nishi-Kobe Medical Center. Of the 1995 responses, 302 were excluded because of missing personal characteristics or items of motivation and hesitation, leaving 1693 (46.7%) questionnaires for analysis. As compared with the distribution of survey respondents key characteristics shown in Table 1, the total staffs of three hospitals had similar proportional distribution, with 73.7% females (compared with 75.7%), 64.2% clinical staffs and 10.3% clinical support/technical staffs (compared with 65.8% and 10.7%), 42.1% 20-30 years old, 27.3% 30-40 years old, 15.6% 40-50 years old and 12.6% 50-60 years old (compared with 36.8%, 27.5%, 19.3%, 14.1%).

**Table 1:** Demographic characteristics of the study population and likelihood of reporting hesitation and motivation to work

				Likelihood of reporting			
				
		High		Hesitation to work		Motivation to work	
**Characteristic**	**n(%)**	**Hesitation n (%)**	**Motivation n (%)**	**Bivariate OR (95%CI)**	**Multivariate**^**+ **^**OR (95%CI)**	**Bivariate OR (95%CI)**	**Multivariate**^**+ **^**OR (95%CI)**

**Age**							
20-30	623 (36.8)	112 (18.0)	111 (17.8)	Reference	Reference	Reference	Reference
30-40	466 (27.5)	86 (18.5)	125 (26.8)	1.0 (0.8-1.4)	1.1 (0.8-1.5)	1.7 (1.3-2.3)	1.6 (1.2-2.1)
40-50	326 (19.3)	29 (8.9)	117 (35.9)	0.4 (0.3-0.7)	0.4 (0.3-0.7)	2.6 (1.9-3.5)	2.3 (1.7-3.2)
50-60	239 (14.1)	20 (8.4)	114 (47.7)	0.4 (0.3-0.7)	0.5 (0.3-0.8)	4.2 (3.0-5.8)	3.4 (2.4-4.8)
60-70	39 (2.3)	2 (5.1)	14 (35.9)	0.2 (0.1-1.0)	0.3 (0.1-1.2)	2.6 (1.3-5.1)	1.8 (0.9-3.7)
**Gender**							
Male	411 (24.3)	38 (9.2)	174 (42.3)	Reference	Reference	Reference	Reference
Female	1282 (75.7)	211 (16.5)	307 (23.9)	1.9 (1.3-2.8)	1.7 (1.2-2.6)	0.4 (0.3-0.5)	0.6 (0.5-0.8)
**Job Classification**							
Clinical staff	1114 (65.8)	180 (16.2)	265 (23.8)	Reference	Reference	Reference	Reference
Clinical technical/Support staff	181 (10.7)	13 (7.2)	81 (44.8)	0.4 (0.2-0.7)	0.6 (0.3-1.0)	2.6 (1.9-3.6)	1.7 (1.2-2.5)
Non-clinical support staff	398 (23.5)	56 (14.1)	135 (33.9)	0.9 (0.6-1.2)	1.2 (0.8-1.7)	1.6 (1.3-2.1)	1.2 (0.9-1.6)
**Working at high risk environment**							
No	1157 (68.3)	169 (14.6)	286 (24.7)	Reference	Reference	Reference	Reference
Yes	536 (31.7)	80 (14.9)	195 (36.4)	1.0 (0.8-1.4)	1.2 (0.9-1.7)	1.7 (1.4-2.2)	1.6 (1.2-2.0)

+ Adjusted for Age, Gender, Job classification, Working place.

Among these 1693 responses, 481 (28.4%) said they were strongly motivated to work and 249 (14.7%) said they were very hesitant to work.

According to the personal characteristics and OR, compared with workers in their 20s, workers in their 30s had higher motivation (Multivariate OR: 1.6; CI 1.2-2.1) without any significant difference in hesitation. Workers in their 40s and 50s had higher motivation (Multivariate OR: 40s; 2.3; CI 1.7-3.2, 50s; 3.4; CI 2.4-4.8) and lower hesitation (Multivariate OR: 40's; 0.4; CI 0.3-0.7, 50's; 0.5; CI 0.3-0.8). Females showed lower motivation (Multivariate OR: 0.6; CI 0.5-0.8) and higher hesitation (Multivariate OR: 1.7; CI 1.2-2.6) than males. Clinical technical/support staff had higher motivation (Multivariate OR: 1.7; CI 1.2-2.5) than clinical staff without any significant difference in hesitation. Working at a high-risk facility was related to higher motivation than working at a low-risk facility (Multivariate OR: 1.6; CI 1.2-2.0) without any significant difference in hesitation (Table 1). The associations between stress-related questions and OR are shown in Table 2. Among the items with significant difference between the responses to the stress-related questions with strong scores and those with weak scores, ORs that are over 2.5 or under 0.4 are indicated as follows; "Being protected by the national or local government" (Multivariate OR: motivation; 3.5; CI 2.2-5.4, hesitation; 0.2; CI 0.1-0.6) and "being protected by hospital" (Multivariate OR: motivation; 2.8; CI 2.2-3.7, hesitation; 0.5; CI 0.3-0.7) were associated with higher motivation and lower hesitation. 94.1% responded that the protection from the national and local governments was weak and 79.7% responded that the protection provided by their hospital was weak. "Elevated mood" was associated with higher motivation without any significant difference in hesitation (Multivariate OR: 4.6; CI 3.3-6.5). The items with higher motivation without any significant difference in hesitation were "burden of child care including lack of nursery" (Multivariate OR: 2.7; CI 1.6-4.5). The items with higher motivation and hesitation were "anxiety about being infected" (Multivariate OR: motivation; 1.3; CI 1.1-1.7, hesitation; 4.8; CI 3.3-7.0), "anxiety about infecting family" (Multivariate OR: motivation; 1.6; CI 1.3-2.0, hesitation; 2.8; CI 2.1-3.8), "anxiety of being infected during commuting" (Multivariate OR: motivation; 1.5; CI 1.2-1.8, hesitation; 2.8; CI 2.1-3.8), "anxiety about compensation" (Multivariate OR: motivation; 1.4; CI 1.1-1.8, hesitation; 3.6; CI 2.7-4.9), "physical exhaustion" (Multivariate OR: motivation; 1.8; CI 1.4-2.3, hesitation; 2.7; CI 2.1-3.6), "mental exhaustion" (Multivariate OR: motivation; 2.6; CI 1.6-4.2, hesitation; 2.7; CI 2.1-3.6), "insomnia" (Multivariate OR: motivation; 2.6; CI 1.6-4.2, hesitation; 2.9; CI 1.7-5.0),and "being isolated" (Multivariate OR: motivation; 1.6; CI 1.0-2.5, hesitation; 4.7; CI 3.0-7.2).

**Table 2:** Associations of stress factors and likelihood of reporting hesitation and motivation to work

					Likelihood of reporting			
					
			Among the strong		Hesitation to work		Motivation to work	
	**Weak n(%)**	**Strong n(%)**	**High Hesitation n (%)**	**High Motivation n (%)**	**Bivariate OR (95%CI)**	**Multivariate**^**+ **^**OR (95%CI)**	**Bivariate OR (95%CI)**	**Multivariate **^**+**^**OR (95%CI)**

**Risk for infection**								
Anxiety about being infected	709 (41.9)	981 (57.9)	212 (21.6)	291 (29.7)	5.2 (3.6-7.4)	4.8 (3.3-7.0)	1.2 (0.9-1.4)	1.3 (1.1-1.7)
Anxiety about infecting family	733 (43.3)	950 (56.1)	191 (20.1)	301 (31.7)	3.0 (2.2-4.0)	2.8 (2.1-3.9)	1.5 (1.2-1.8)	1.6 (1.3-2.0)
Anxiety of being infected during commuting	905 (53.5)	781 (46.1)	169 (21.6)	235 (30.1)	2.9 (2.2-3.9)	2.8 (2.1-3.8)	1.2 (0.9-1.4)	1.5 (1.2-1.8)
**Knowledge and measurement**								
Lack of knowledge about infectiosity and virulence	1017 (60.1)	666 (39.3)	132 (19.8)	214 (32.1)	1.9 (1.5-2.5)	1.8 (1.4-2.4)	1.4 (1.1-1.7)	1.5 (1.2-1.9)
Lack of knowledge about prevention and protection	1337 (79.0)	348 (20.6)	88 (25.3)	107 (30.7)	2.5 (1.9-3.3)	2.3 (1.7-3.1)	1.1 (0.9-1.5)	1.3 (1.0-1.7)
**Protection**								
Feeling of being protected by country and local government	1593 (94.1)	96 (5.7)	3 (3.1)	56 (58.3)	0.2 (0.1-0.5)	0.2 (0.1-0.6)	3.9 (2.5-5.9)	3.5 (2.2-5.4)
Feeling of being protected by hospital	1349 (79.7)	338 (20.0)	26 (7.7)	168 (49.7)	0.4 (0.3-0.6)	0.5 (0.3-0.7)	3.3 (2.6-4.2)	2.8 (2.2-3.7)
Anxiety about compensation	906 (53.5)	780 (46.1)	183 (23.5)	240 (30.8)	3.9 (2.9-5.3)	3.6 (2.7-4.9)	1.2 (1.0-1.5)	1.4 (1.1-1.8)
**Condition**								
Burden of increase quantity of work	1098 (64.9)	589 (34.8)	107 (18.2)	192 (32.6)	1.5 (1.1-2.0)	1.6 (1.2-2.1)	1.4 (1.1-1.7)	1.2 (0.9-1.5)
Burden of change of quality of work	1096 (64.7)	592 (35.0)	101 (17.1)	201 (34.0)	1.4 (1.0-1.8)	1.4 (1.0-1.9)	1.5 (1.2-1.9)	1.4 (1.1-1.8)
Physical exhaustion	1151 (68.0)	541 (32.0)	124 (22.9)	187 (34.6)	2.4 (1.9-3.2)	2.5 (1.8-3.3)	1.5 (1.2-1.9)	1.5 (1.2-1.9)
Mental exhaustion	1122 (66.3)	565 (33.4)	134 (23.7)	202 (35.8)	2.7 (2.1-3.6)	2.7 (2.1-3.6)	1.7 (1.4-2.1)	1.8 (1.4-2.3)
Insomnia	1618 (95.6)	73 (4.3)	22 (30.1)	38 (52.1)	2.6 (1.6-4.4)	2.9 (1.7-5.0)	2.9 (1.8-4.6)	2.6 (1.6-4.2)
Elevated mood	1505 (88.9)	185 (10.9)	35 (18.9)	115 (62.2)	1.4 (0.9-2.1)	1.6 (1.0-2.4)	5.1 (3.7-7.1)	4.6 (3.3-6.5)
**Isolation**								
Feeling of being avoided by others	1495 (88.3)	192 (11.3)	56 (29.2)	44 (22.9)	2.8 (2.0-4.0)	2.4 (1.7-3.4)	0.7 (0.5-1.0)	0.9 (0.6-1.3)
Feeling of being isolated	1588 (93.8)	104 (6.1)	42 (40.4)	38 (36.5)	4.5 (3.0-6.9)	4.7 (3.0-7.2)	1.5 (1.0-2.3)	1.6 (1.0-2.5)
**Others**								
Feeling of having no choice but to work due to obligation	604 (35.7)	1081 (63.9)	186 (17.2)	330 (30.5)	1.9 (1.4-2.6)	1.7 (1.3-2.4)	1.4 (1.1-1.7)	1.6 (1.2-2.0)
Burden of child care including lack of nursery	380 (22.4)	221 (13.1)	56 (25.3)	74 (33.5)	3.0 (1.9-4.7)	2.7 (1.6-4.5)	1.0 (0.7-1.4)	1.3 (0.9-1.9)

+ Adjusted for Age, Gender, Job classification, Working place.

The percentage of workers that considered childcare to be a burden was significantly higher among females (43.2%) than males (21.3%).

## Discussion

Although some studies have examined professionalism or willingness to work in a hypothetical pandemic or high-risk infection and one study examined the hospital absentee rate during an actual H1N1 pandemic [20], as far as we know, our survey is the only one that evaluated hospital workers' willingness to work and the influencing factors following an actual pandemic infection. Our study was focused on the factors associated with willingness. The results show that willingness has conflicting aspects. That is, factors that raise motivation do not necessarily lower hesitation: some factors raise both motivation and hesitation.

We found factors were categorised into four types according to their influence on the OR of motivation and hesitation to work. That is, some factors increased the OR of motivation and lowered the OR of hesitation, other factors increased the OR of motivation only, other factors increased the OR of hesitation only, and others increased the OR of both motivation and hesitation. This is important because understanding factors that cause or reduce conflict is necessary to find ways to support professionalism of hospital workers in a high-risk environment.

A limitation of our study is the non-response bias as a result of the 46.7% response rate. However, the total number of subjects was large and their demographics to the population as a whole that did not make noticeable difference.

The most important factors are ones that resolve conflicting emotions and promote willingness, that is, increase motivation and lower hesitation. Above all, the various types of protection that workers receive from the national and local governments and from their hospitals (e.g. protecting them from getting sick and from malpractice suits) needs improvement. The physicians, nurses and others in the ward for H1N1 and the outpatient department for H1N1were provided with protection suits, N95 masks, goggles and antiviral prophylaxes but many of them felt that they were not strongly protected by the national and local government and hospitals. There were no plans about what they should do or how they would be reimbursed in case they became infected and the governments provided no encouraging words to the hospitals. In a study of the use of the antiviral oseltamivir as a prophylactic [21], 274 employees who worked in high risk places at Kobe City Medical Center General Hospital (KCGH) took oseltamivir from May 16 to May 25, 2009. Only 37% took the medicine for the full ten days. The others stopped taking it for a variety of reasons, including side effects, anxiety about the drug, failure to remember taking it, or because the virulence of H1N1 seemed weak.

The fact that governmental and hospital protection increased motivation and lowered hesitation suggests that positive intervention in these fields will have the strongest impact on reducing non-illness-related absenteeism. Therefore, the protection of hospital workers by governments and hospitals should be emphasized [22-24]. Samuel et al. [25] suggested that two major factors are involved in instilling employees sense of ethical obligations to treat patients during a crisis. First is an expectation of some reciprocal social obligations. For example, in preparation for epidemics, communities or employers should take all reasonable precautions to prevent illness among health care workers and their families, provide for the care of those who do become ill, reduce or eliminate malpractice threats for those working in high-risk emergency situations and provide reliable compensation for the families of those who die while fulfilling this duty. Second, the duty of physicians should be attenuated but not eliminated, by his or her responsibility in order to prevent them from becoming patients [25]. Work can be attenuated by reducing working time, by restricting the number of patients, by assigning physician to a place with lower workload or by shifting them to jobs with lower risk. In order for workers to fulfil their duties, they need to feel safe. The feeling of safety will be strong when the safety is provided by their organizations. But, in addition to these measurements, there is a need for frequent communication between individual workers and their organization or governments. Encouragement from organizations or governments would also support workers mentally.

In the present study, increased motivation and less hesitation was noted in middle-aged and male workers. Age and gender were also examined in two studies that presented hospital workers with a hypothetical influenza pandemic in the United States [3] and a hypothetical SARS pandemic in Singapore [26]. These studies found no age or gender difference in the willingness to work, which is inconsistent with our results. This discrepancy may be partly because people in management positions have a strong sense of responsibility, and in our hospitals, many of the management positions are held by males in their 40s and 50s. Another reason for the discrepancy is that our study was based on a real pandemic and the others were based on hypothetical pandemics. As for gender, studies of physicians' burnout have indicated that females feel more stress than males in the workplace [27,28]. As a result, extra measures should be taken to alleviate the stress of female workers during stressful events, such as by providing childcare services.

Factors that increase motivation only may not always be good because they could result in overfatigue in the long run. Paradoxically, we found that working in a place of high risk and demands for unaccustomed work increased motivation. A Canadian study of senior practitioners with reputations for resilience indicated that making a unique contribution, and receiving privileges and rewards are central to building resilience, although the burden of increased workload was found to lower the level of satisfaction [29]. In view of these results, working in a place of high risk with new work may be considered as a special contribution by hospital workers. Technical/support staffs were especially motivated, perhaps because, in addition to the above reason, they usually had little direct contact with patients and thus had lower perceived levels of risk.

Reducing the factors that cause hesitation only will reduce the barrier to work in high-risk situations. Such stress factors include a lack of knowledge about prevention and protection, the burden of increased quantity of work, the feeling of being avoided by others, and the burden of childcare without childcare facilities. Examples of such measures include work sharing or rotation of duty. Sharing of duties and increasing the number of people who work in high-risk places will provide workers with more concrete knowledge about prevention and protection, lighten their workload, promote a sense of unity and reduce the sense of isolation.

Reducing factors that increase both motivation and hesitation should be given high priority, as these factors can result in the conflict among hospital workers in the long term, although in the short term they may cancel each other out. In the present study, many of the respondents had strong fears of being infected (57.9% of respondents), infecting family (56.1%), feeling of having no choice but to work due to obligation (63.9%) and anxiety about compensation in case of being infected (53.5%). During an infection pandemic, it is to some degree inevitable to feel exhausted and isolated and to worry about becoming infected. But a study said that mitigation strategies that include options for preferential access to either antiviral therapy, protective equipment, or both for the employee as well as his or her immediate family will have the greatest impact [30]. Our hospitals provided all protective measurements listed above to the employee but not to his or her immediate family. The measurement should include protection of employees' family, which might support their motivation and reduce hesitation. In addition, government and hospital managers should develop plans to compensate and treat workers that become infected and to help workers meet their obligations. This would also increase the feeling of protection given by the hospital and the various levels of government.

Although our survey was related to an influenza pandemic, most of the questions used here have generalizability to other high-risk situations. Further studies are needed to test the external validity of our results.

## Conclusions

We found that there are factors which influence motivation and hesitation to work in an influenza pandemic. Some factors have conflicting effects that increase both motivation and hesitation. Giving workers the feeling that they are being protected by the national and local governments and by their hospital is especially valuable because it increases their motivation and lowers their hesitation to work. This can be achieved by not only providing protective materials and compensation but also by frequently communicating with and encouraging workers.

We should prepare for severer and longer infection pandemic as soon as possible.

## Competing interests

The authors declare that they have no competing interests.

## Authors' contributions

HI was the lead writer and worked on the content development and distribution of the survey instrument. KM coordinated the three hospitals survey protocol. AI, KM and NK developed survey instrument content and distributed of the survey instrument at Kobe City Medical Center General Hospital. KA and KM, and AK, MI and ST distributed the survey instrument and cooperated data analysis at Kobe City Medical Center West Hospital and West Kobe Medical Center respectively. TM provided guidance on whole process. All authors read and approved the final manuscript.

## Pre-publication history

The pre-publication history for this paper can be accessed here:

http://www.biomedcentral.com/1471-2458/10/672/prepub

## Supplementary Material

Additional file 1 Questionnaire Click here for file
