# Supplementary material for: Factors associated with motivation and hesitation to work among health professionals during a public crisis: a cross sectional study of hospital workers in Japan during the pandemic (H1N1) 2009
Source: BMC Public Health. 2010 Nov 4;10:672. doi: 10.1186/1471-2458-10-672 (PMC3091577; doi:10.1186/1471-2458-10-672)
Supplement: Additional file 1 — Questionnaire [file 1471-2458-10-672-S1.DOC]

To Mr. and Ms. Hospital workers;

Thank you very much for your notice.

We, the department of psychiatry and nursing, are administrating **research about stresses accompanied with H1N1 influenza pandemic**.

To maintain hospital function in the emergent situation, it is important to know what caused the stress during this pandemic infection.

We are sorry for bothering you a lot, but please answer the questions below.

The results will be reported to you all, presented in the associated society and submitted to the related journals.

This is anonymous questionnaire and the answers will be analyzed as digit number, so individuals can’t be identified.

**The questions start from here**

Please circle the items which you think are appropriate

Do you approve that your answers will be used and the results be published ?　: Yes No

**Age**　：　1. 20’s　2. 30’s　3. 40’s　4. 50’s　5. 60’s

**Gender**　：　1. Male 　2. Female

**Job types**：1. Doctor　2.Nurse　3. Office worker　4. Radiological technician　5. Laboratory technician　6. Pharmacist　7. Dietician　8. Social worker　9. Physical therapist, Occupational therapist or Speech therapist　10. Clinical clerk　11. Guard person　12. Janitor

13. Others ( 　　　　　　）

**Working place（multiple answers allowed）**：

1. Ward for the H1N1 influenza infection 2. The outpatient department for H1N1 influenza infection 3. Emergency outpatient unit 4. Headquarter 5. Others ( )

“Stresses and symptoms associated work during H1N1 influenza pandemic”

Please recall your work during H1N1 influenza pandemic and answers questions below

|  |  | 0 Never | 1 Rarely | 2 Sometimes | 3 Always |
| --- | --- | --- | --- | --- | --- |
| 1 | I felt anxious about being infected |  |  |  |  |
| 2 | I felt anxious about infecting family |  |  |  |  |
| 3 | I felt burden of increased quantity of work |  |  |  |  |
| 4 | I felt burden of changed quality of work |  |  |  |  |
| 5 | I felt anxious about being infected during commuting |  |  |  |  |
| 6 | I felt lack of knowledge about prevention and protection |  |  |  |  |
| 7 | I felt lack of knowledge about infectiosity and virulence |  |  |  |  |
| 8 | I felt I was avoided by others |  |  |  |  |
|  |  | 0 Never | 1 Rarely | 2 Sometimes | 3 Always |
| 9 | I felt I was protected by country or local government |  |  |  |  |
| 10 | I felt I was protected by my hospital |  |  |  |  |
| 11 | I felt anxious about compensation in the case of being infected. |  |  |  |  |
| 12 | I felt hesitated to work. |  |  |  |  |
| 13 | I felt I was isolated |  |  |  |  |
| 14 | I felt elevated mood |  |  |  |  |
| 15 | I had insomnia |  |  |  |  |
| 16 | I was exhausted physically |  |  |  |  |
| 17 | I was exhausted mentally |  |  |  |  |
| 18 | I felt motivated to work |  |  |  |  |
| 19 | I felt I had no choice but to work due to obligation |  |  |  |  |

If you have a child or children, please answer the question below;

| 20 | I felt burden of increasing child care including lack of nursery |  |  |  |  |
| --- | --- | --- | --- | --- | --- |
